# Supplementary material for: Lying in wait: the resurgence of dengue virus after the Zika epidemic in Brazil
Source: Nat Commun. 2021 May 11;12:2619. doi: 10.1038/s41467-021-22921-7 (PMC8113494; doi:10.1038/s41467-021-22921-7)
Supplement: Supplementary file 1 — Supplementary Information [file 41467_2021_22921_MOESM1_ESM.pdf]

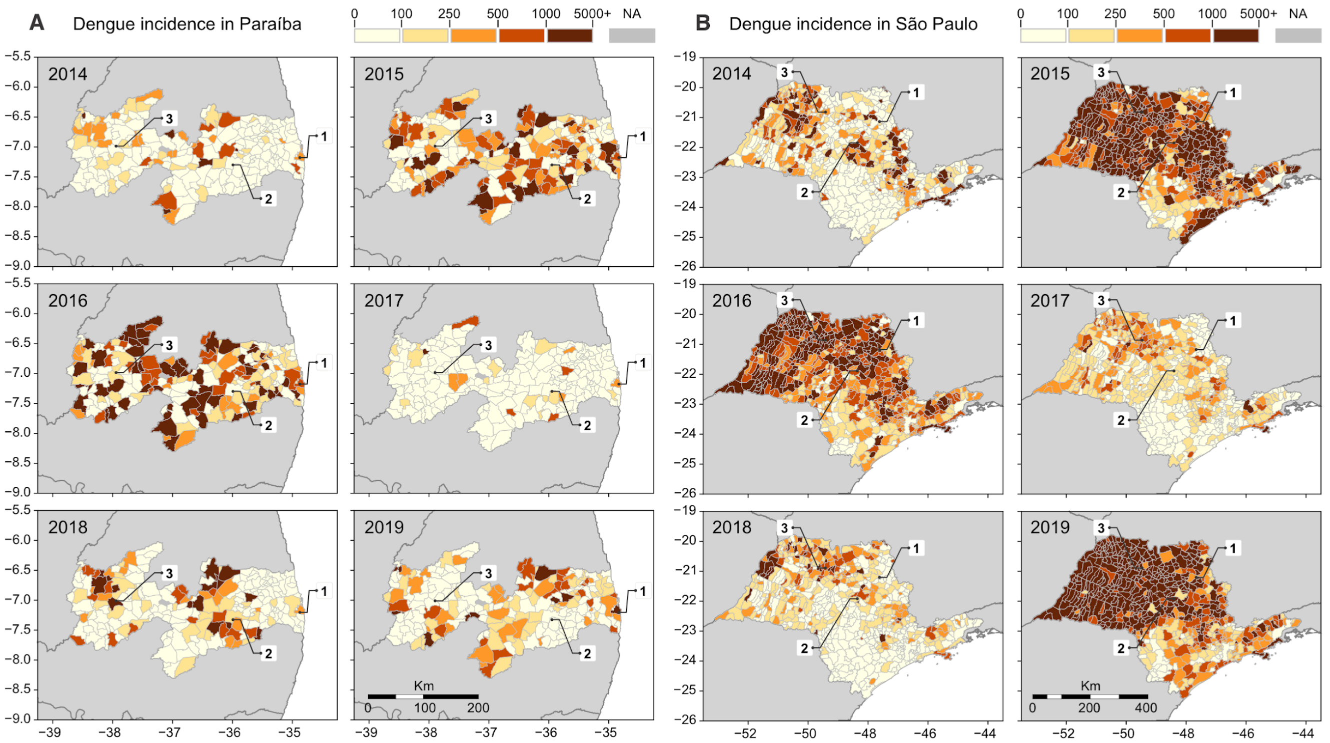

Figure S1. Dengue incidence (per 100,000 population) per municipality in Paraíba and São Paulo states, 2014-2019. The incidence of dengue in Paraíba (A) and São Paulo (B) between 2014 and 2019 shows a pattern that resembles that of cases throughout Brazil, with year of low incidence in most municipalities, such as in 2014 and 2017, and years of much high incidence, like 2015, 2016 and 2019. While the 2018 incidence in São Paulo was similar to that of 2017, in Paraíba the dengue incidence in 2018 reveals an early surge of dengue in that state, similar to that observed in 2019. As in Figure 2, the three main cities included in our study are highlighted in each state. In Paraíba (A) we show: 1. João Pessoa; 2. Campina Grande; and 3. Coremas. In São Paulo (B) we highlight: 1. Ribeirão Preto; 2. Araraquara; and 3. São José do Rio Preto.

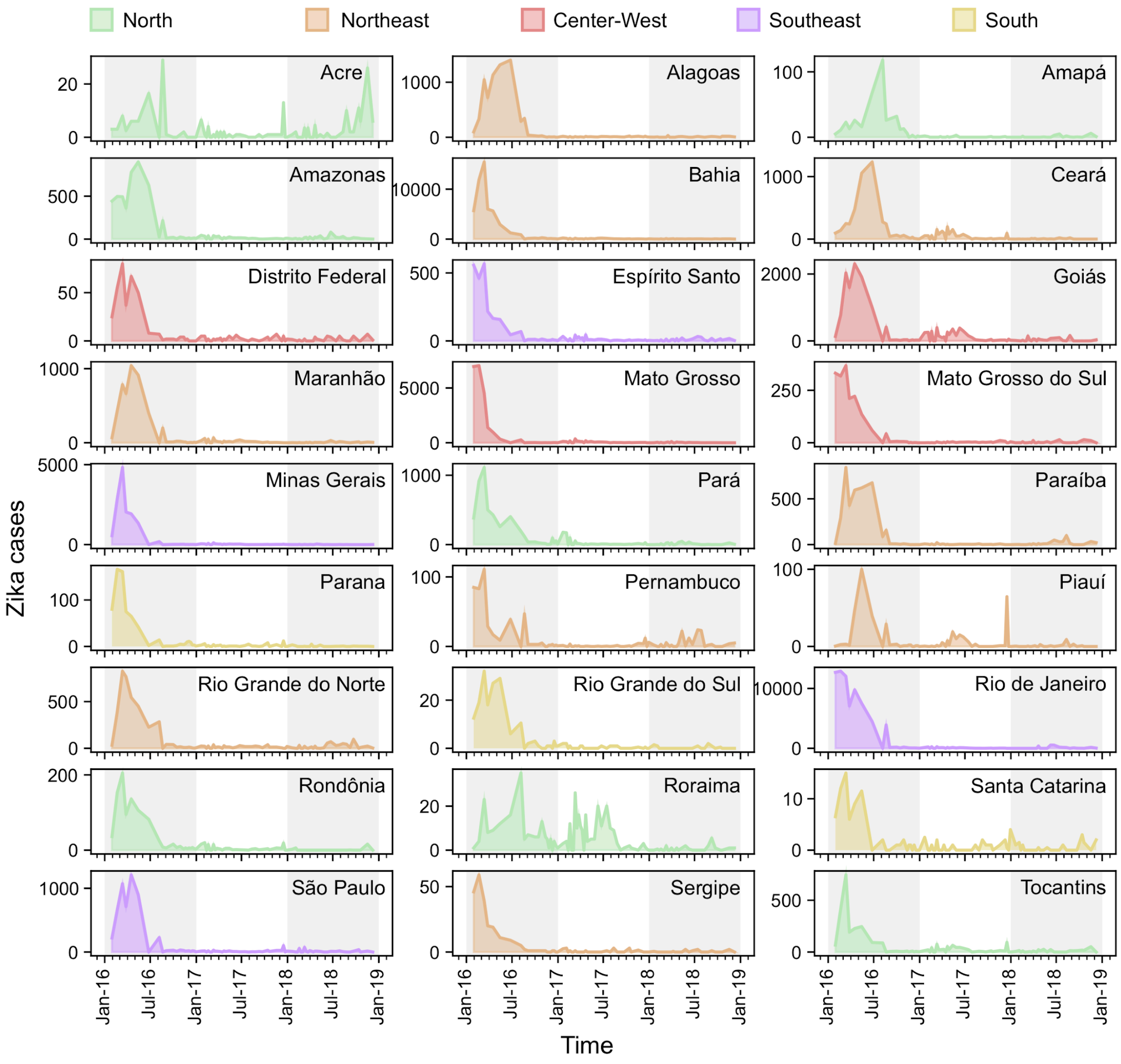

Figure S2. Zika cases in Brazil per state, 2016-2018. Most states experienced a peak in Zika cases between February and July 2016.

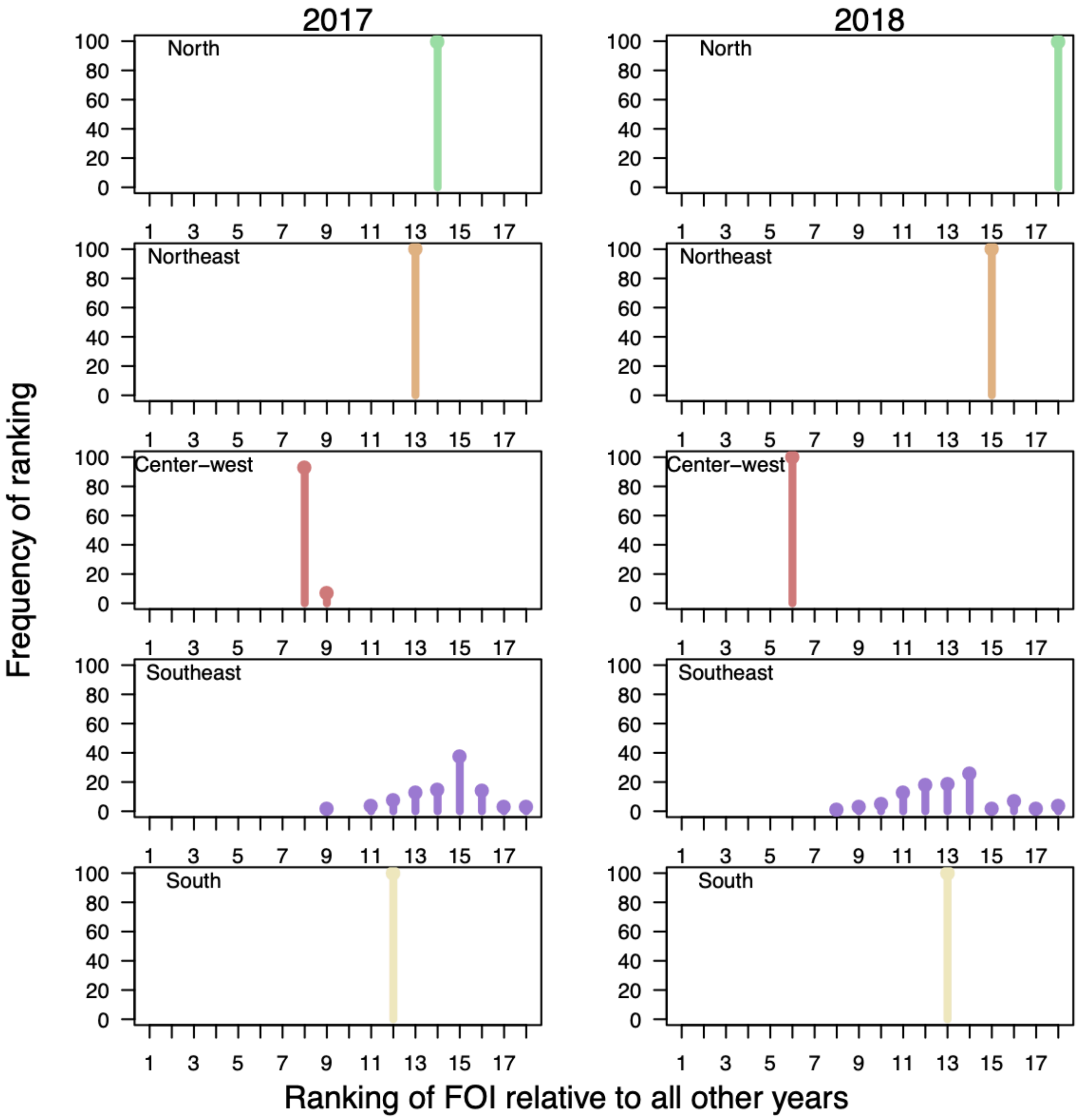

Figure S3. Ranking of annual force of infection estimates across years between 2002 and 2019. Ranking 100 maximum-likelihood estimates of Force of Infection (FOI) for each region in Brazil, for the two recent years of low dengue incidence (2017-2018) in the left and right columns, respectively.

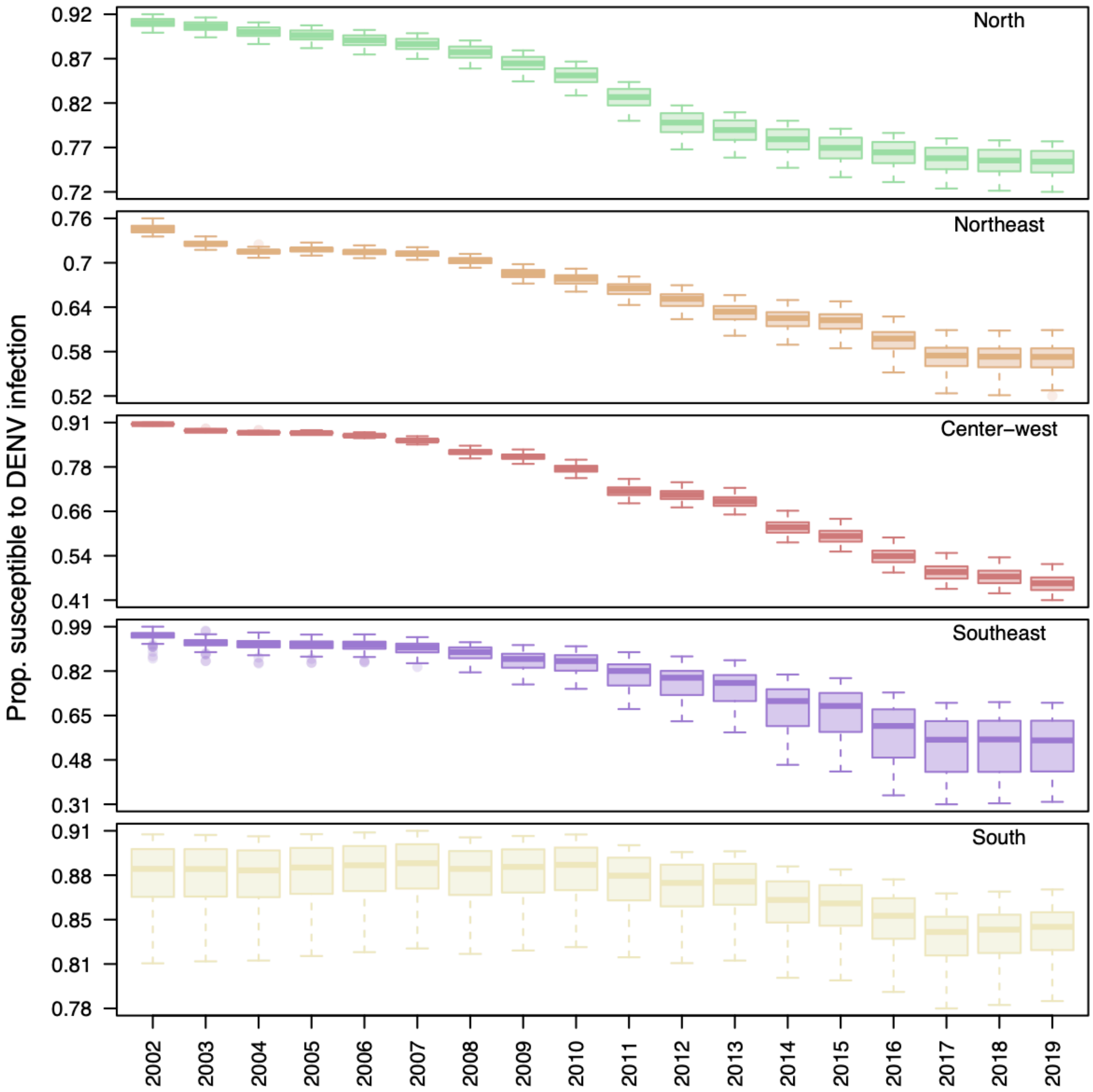

Figure S4. Proportion susceptible to DENV. Annual proportions of susceptible population per geographic region in Brazil, from 2002 to 2019. Regions are ordered (top to bottom) by North (green), Northeast (orange), Center-west (red), Southeast (purple), and South (tan) with colors corresponding to Figures 1 and 3. Values displayed here come from the set of 100 final estimates, which reflect the top 10% of 1,000 parameter sets. These originally involved sampling $\vec{\gamma}_F$ and $\vec{\gamma}_S$ from assumed uniform priors (specified in **Table S1**) and then finding maximum-likelihood estimates of other model parameters. Boxes indicate the interquartile ranges and whiskers indicate the 95% quantiles of those 100 points, with dark lines representing median estimates.

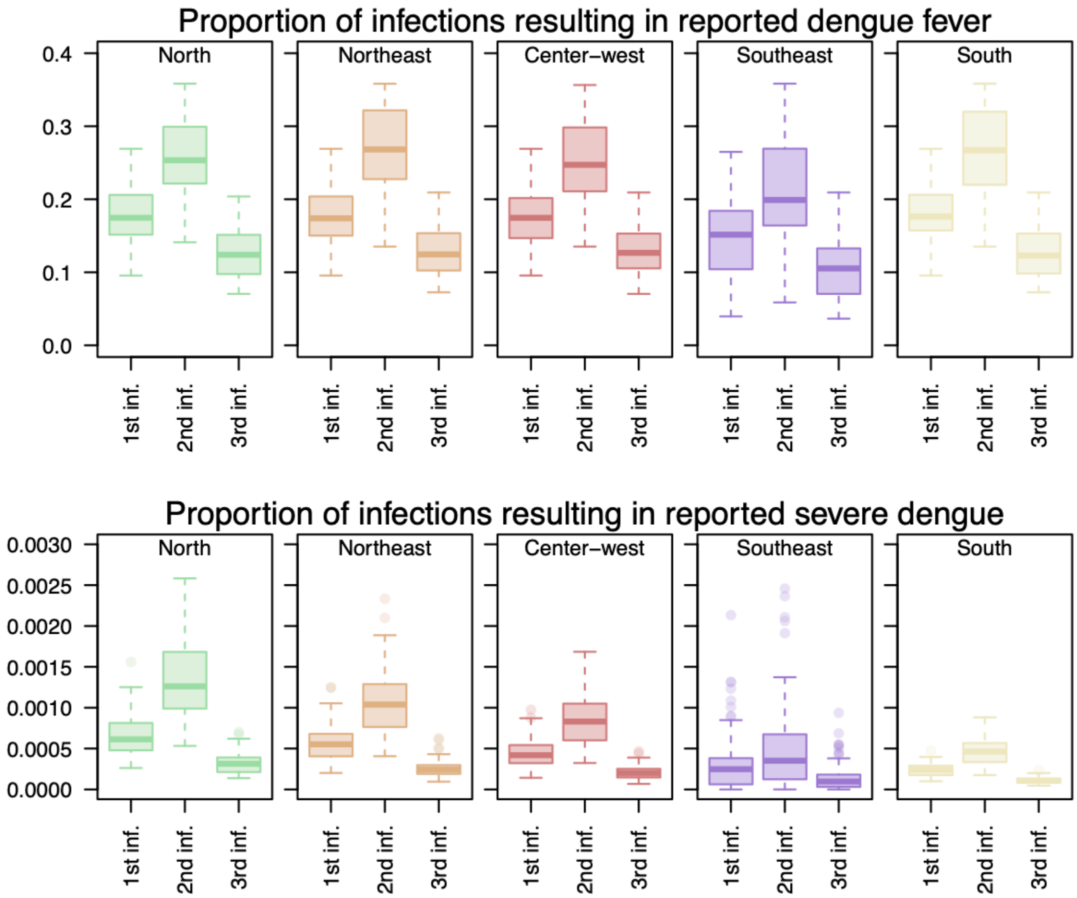

Figure S5. Proportion of infections resulting in reported dengue fever (top) or severe dengue (bottom). These represent combined probabilities of $\varrho_{F,r}$, $\varrho_{S,r}$, $\vec{\gamma}_F$, and $\vec{\gamma}_S$ dependent on whether an individual is experiencing their first, second, or third infection. Regions are ordered (left to right) by North (green), Northeast (orange), Center-west (red), Southeast (purple), and South (tan) with colors corresponding to Figures 1 and 3. Values displayed here come from the set of 100 final estimates, which reflect the top 10% of 1,000 parameter sets. These originally involved sampling $\vec{\gamma}_F$ and $\vec{\gamma}_S$ from assumed uniform priors (specified in **Table S1**) and then finding maximum-likelihood estimates of other model parameters. Boxes indicate the interquartile ranges and whiskers indicate the 95% quantiles of those 100 points, with dark lines representing median estimates.

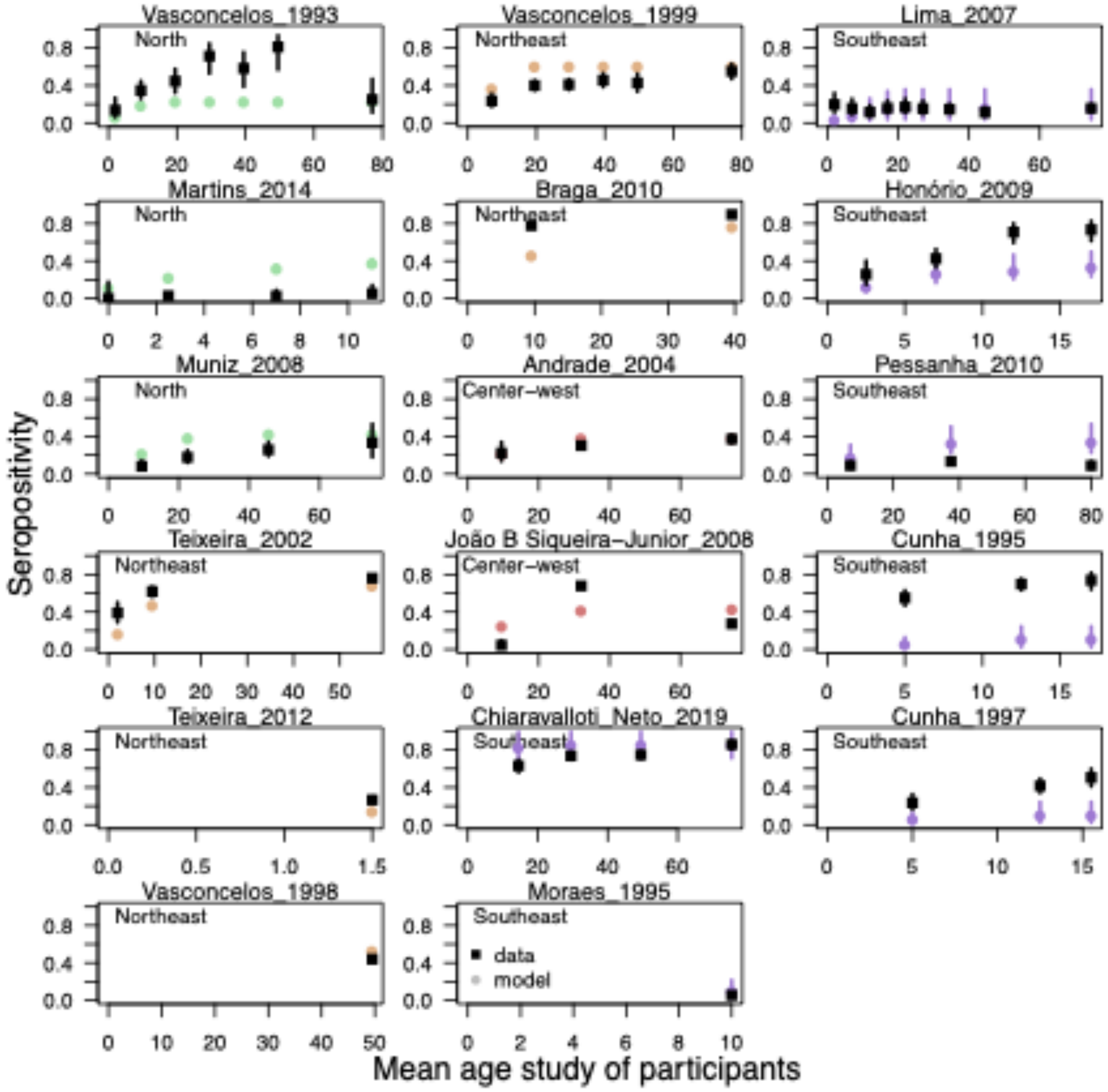

Figure S6. Correspondence between model-estimated seropositivity (colored circles) and empirical estimates (black squares) of seropositivity from seroprevalence studies. Lines indicate 95% credible intervals, with uncertainty for empirical estimates quantified using a beta-binomial conjugate prior relationship that assumed uniform priors. In general, there was good agreement between model fits and empirical estimates, although in some cases model predictions were lower or higher. This could be a result of discrepancies between the forces of infection implied by serological data and those implied by reported case data. One reason that might occur in some instances is because serological studies tended to be relatively localized and were not necessarily representative of an entire region of the country. Studies are referenced on top of each panel, with additional information about these studies provided in **Table S3**. Regions included are North (green), Northeast (orange), Center-west (red), and Southeast (purple) with colors corresponding to Figures 1 and 3.

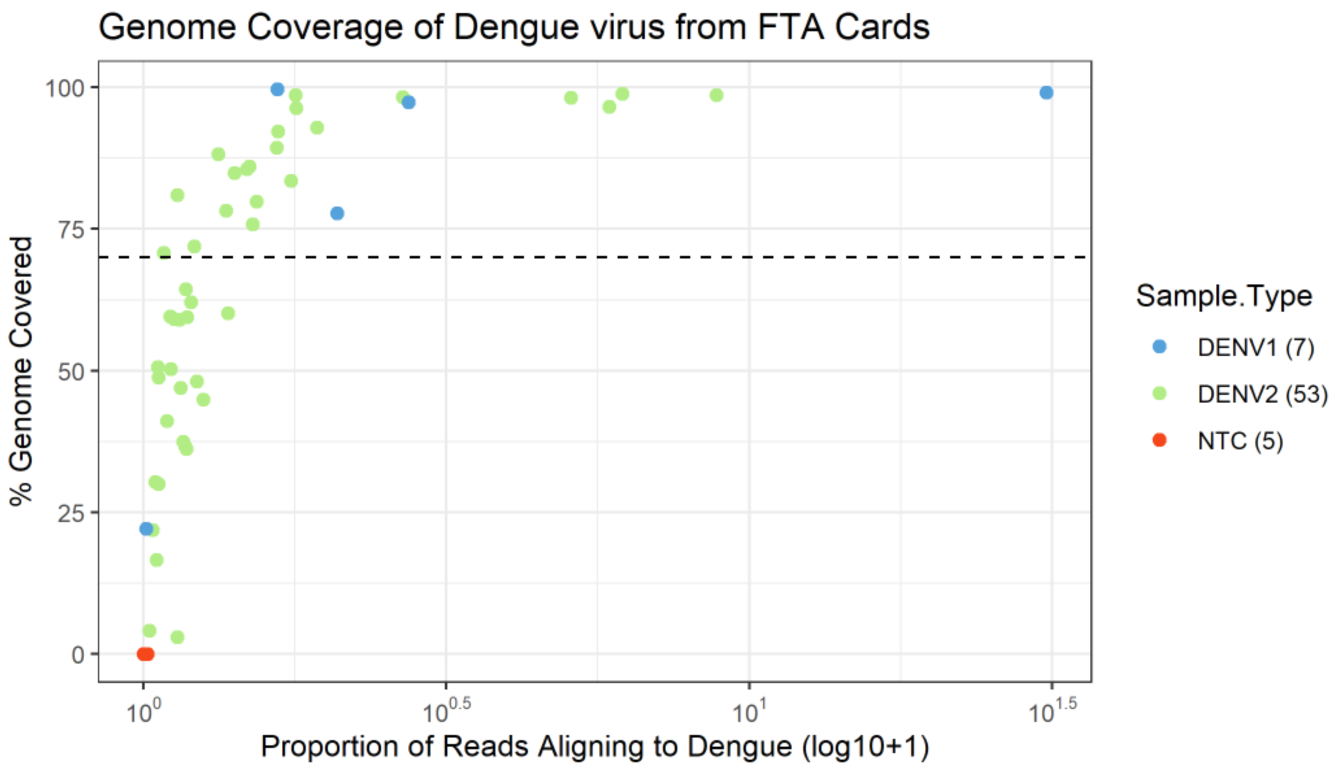

Figure S7. Genome coverage of DENV-1 and DENV-2 samples sequenced from FTA filter paper cards. Dotplot showing the percentage of the genome covered by >10X depth by the overall proportion of reads aligning to the DENV genome generated. Dashed line represents a 70% genome coverage threshold. NTC = No template control ("Water control").

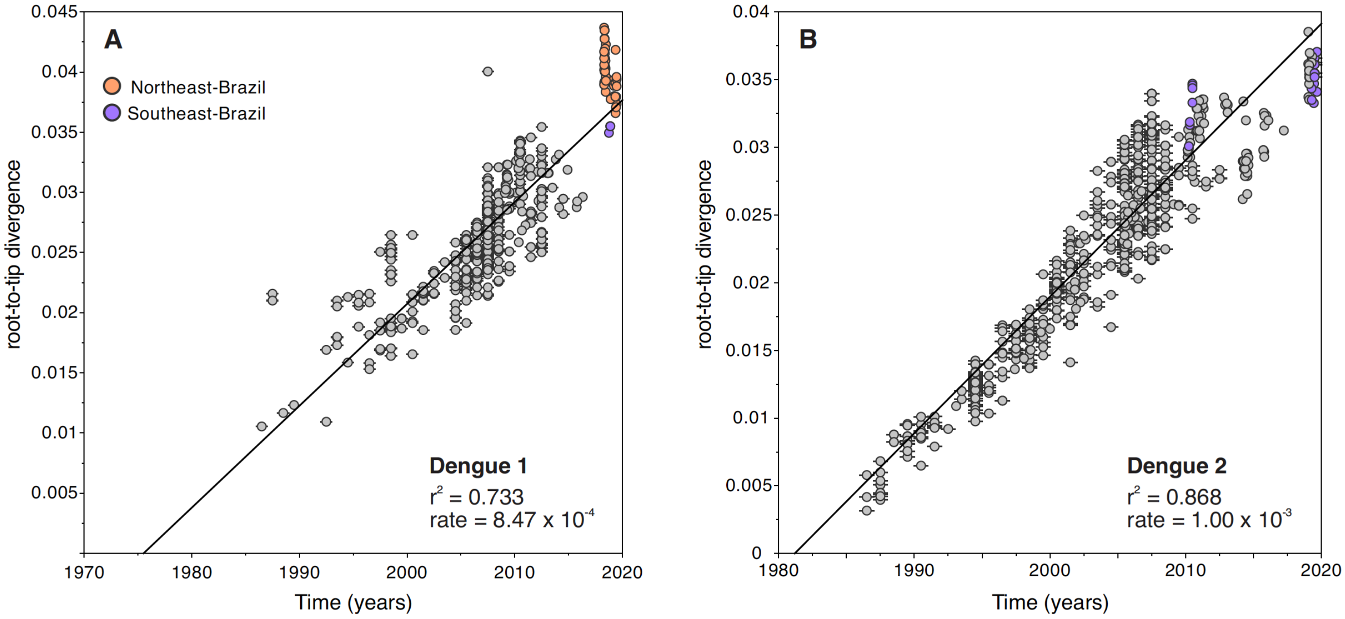

Figure S8. Root-to-tip analyses of DENV-1 and DENV-2 genomes used in phylogenetic analyses. These plots were obtained using TempEst, using ML phylogenies obtained from complete genomes of (**A**) DENV-1 (n=458) and (**B**) DENV-2 (n=700). They show the correlation between the collection time (years) and the genetic divergence (substitutions per site) from the root of the tree (Most Recent Common Ancestors, MRCA) to the tips (sampled genomes). The slope represents the evolutionary rate: 8.47×10^{-4} substitutions/site/year for DENV-1; and 1×10^{-3} substitutions/site/year for DENV-2. All the newly sequenced genomes generated in this study fit the expected molecular clock of viruses from their serotypes.

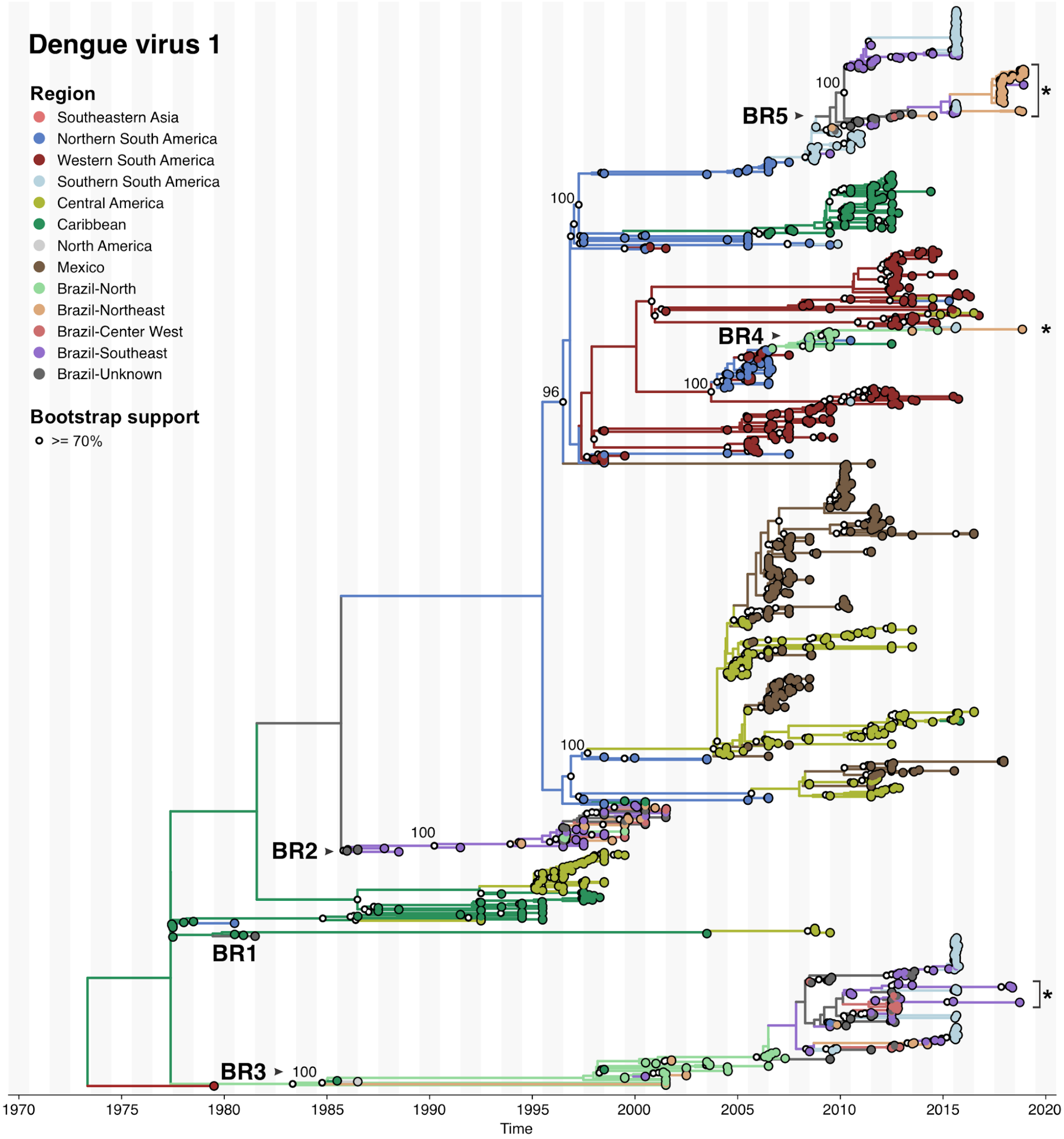

Figure S9. Maximum-likelihood phylogeographic reconstruction of DENV-1 evolution in the Americas using envelope sequences. A total of 1250 envelope sequences were included in this analysis (new genomes highlighted with asterisks), performed using the augur pipeline from Nextstrain. BR2-BR5 represent lineages of DENV-1, numbered in sequential order based on their dates of introduction in Brazil, as previously described[50]. DENV-1 genomes sequenced in this study (n=46) are highlighted with asterisks (*). Data visualization was obtained using baltic.py. An interactive version of this phylogeographic reconstruction and metadata can be found at https://nextstrain.org/community/grubaughlab/DENV-genomics/DENV1-Brazil.

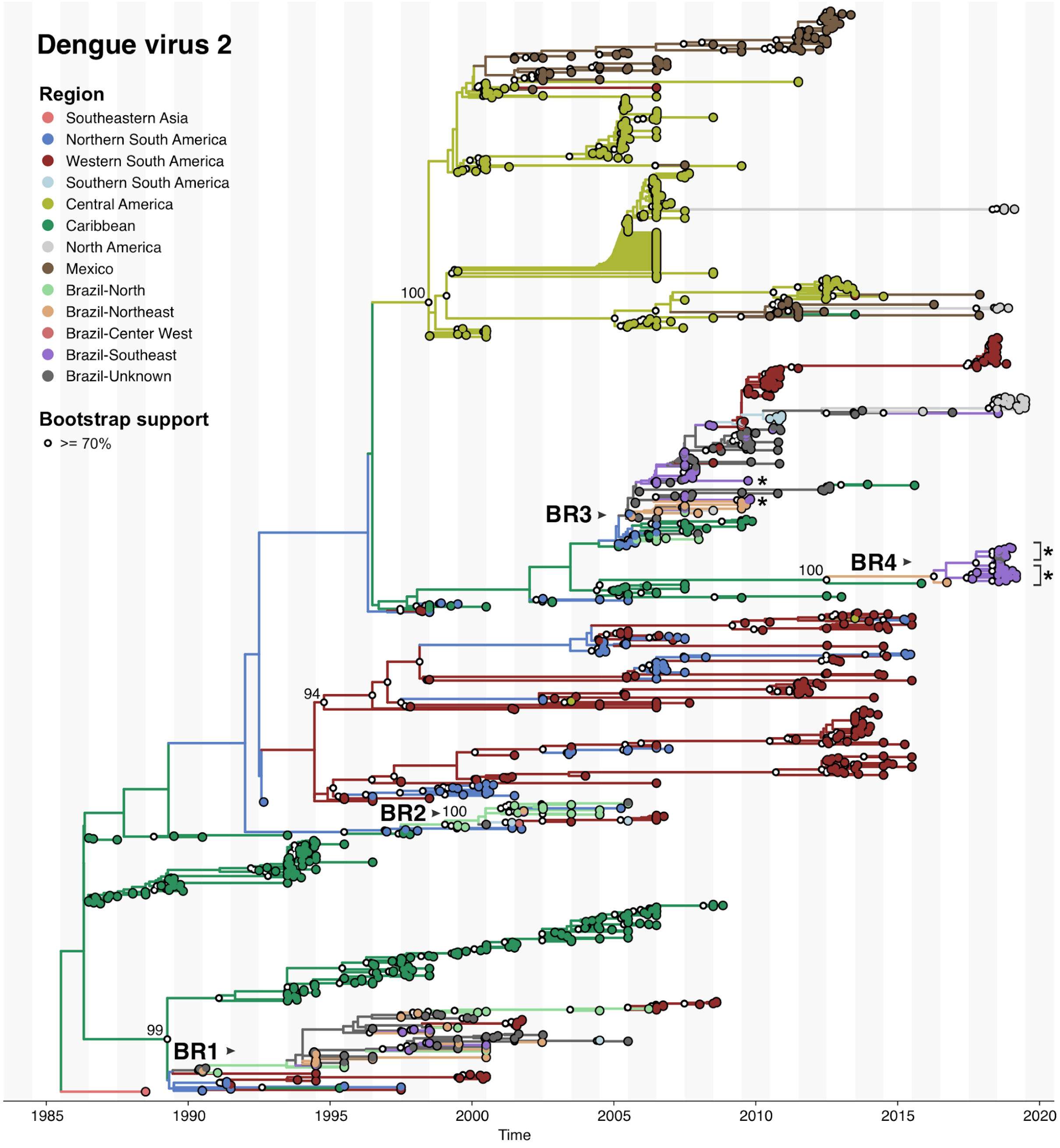

Figure S10. Maximum-likelihood phylogeographic reconstruction of DENV-2 evolution in the Americas using envelope sequences. A total of 1202 envelope sequences were included in this analysis (new genomes highlighted with asterisks), performed using the augur pipeline from Nextstrain. BR1-BR4 represent lineages of DENV-1, numbered in sequential order based on their dates of introduction in Brazil, as previously described [49,52]. DENV-2 genomes sequenced in this study (n=23) are highlighted with asterisks (*). Data visualization was obtained using baltic.py. An interactive version of this phylogeographic reconstruction and metadata can be found at https://nextstrain.org/community/grubaughlab/DENV-genomics/DENV2-Brazil.

Table S1. Parameter ranges for $\vec{\gamma}_F$ and $\vec{\gamma}_S$ used in estimating force of infection. The midpoints of $\vec{\gamma}_F$ were taken from Perkins et al. (Perkins et al., 2019), and the midpoints of $\vec{\gamma}_S$ were taken from Flasche et al. (Flasche et al., 2016). Minimum and maximum values of the range explored are 50% lower and higher, respectively, than the midpoint.

Parameter	Minimum	Midpoint	Maximum
γ_F^0	0.09	0.18	0.36
γ_F^1	0.12	0.24	0.48
γ_F^{2+}	0.07	0.14	0.28
γ_S^0	0.055	0.111	0.222
γ_S^1	0.105	0.209	0.418
γ_S^{2+}	0.026	0.052	0.104

Table S2. List of new DENV-1 (n=46) and DENV-2 (n=23) genomes sequenced and included in this study.

strain	sample name	accession number	virus	genotype	date
USP-CB-111	USP-CB-111	MW208056	Dengue virus 1	V	2019-06-05
USP-CB-51	USP-CB-51	MW208042	Dengue virus 1	V	2018-10-01
USP-CB-53	USP-CB-53	MW208043	Dengue virus 1	V	2018-11-11
USP-CB-54	USP-CB-54	MW208044	Dengue virus 1	V	2018-11-18
USP-CB-103	USP-CB-103	MW208053	Dengue virus 2	AA	2019-09-05
USP-CB-104	USP-CB-104	MW208054	Dengue virus 2	AA	2019-09-05
USP-CB-110	USP-CB-110	MW208055	Dengue virus 2	AA	2019-05-21
USP-CB-113	USP-CB-113	MW208057	Dengue virus 2	AA	2019-06-12
USP-CB-116	USP-CB-116	MW208058	Dengue virus 2	AA	2019-06-19
USP-CB-121	USP-CB-121	MW208059	Dengue virus 2	AA	2019-07-11
USP-CB-123	USP-CB-123	MW208060	Dengue virus 2	AA	2019-07-18
USP-CB-45	USP-CB-45	MW208041	Dengue virus 2	AA	2018-04-17
USP-CB-64	USP-CB-64	MW208045	Dengue virus 2	AA	2019-01-19
USP-CB-67	USP-CB-67	MW208046	Dengue virus 2	AA	2019-01-28
USP-CB-7	USP-CB-7	MW208040	Dengue virus 2	AA	2018-02-19
USP-CB-70	USP-CB-70	MW208047	Dengue virus 2	AA	2019-01-29
USP-CB-74	USP-CB-74	MW208048	Dengue virus 2	AA	2019-01-25
USP-CB-87	USP-CB-87	MW208049	Dengue virus 2	AA	2019-03-12
USP-CB-95	USP-CB-95	MW208050	Dengue virus 2	AA	2019-04-09
USP-CB-96	USP-CB-96	MW208051	Dengue virus 2	AA	2019-04-18
USP-CB-98	USP-CB-98	MW208052	Dengue virus 2	AA	2019-04-16
USP-HC-31	USP-HC-31	MW208062	Dengue virus 2	AA	2019-04-05
USP-HC-37	USP-HC-37	MW208063	Dengue virus 2	AA	2019-04-11
USP-CB-173	USP-CB-173	MW208061	Dengue virus 2	AA	2010-03-26
USP-LC-158	USP-LC-158	MW208064	Dengue virus 2	AA	2010-03-27
USP-LC-269	USP-LC-269	MW208065	Dengue virus 2	AA	2010-04-22
USP-LC-312	USP-LC-312	MW208066	Dengue virus 2	AA	2010-04-23
FIOCRUZ-0017	PB-644	MT862895	Dengue virus 1	V	2018-04-20
FIOCRUZ-0011	PB-635	MT862893	Dengue virus 1	V	2018-04-21
FIOCRUZ-0014	PB-639	MT862891	Dengue virus 1	V	2018-04-21
FIOCRUZ-0016	PB-641	MT862862	Dengue virus 1	V	2018-04-21
FIOCRUZ-0012	PB-636	MT862890	Dengue virus 1	V	2018-04-22
FIOCRUZ-0015	PB-640	MT862894	Dengue virus 1	V	2018-04-22
FIOCRUZ-0013	PB-638	MT862883	Dengue virus 1	V	2018-04-23
FIOCRUZ-0019	PB-706	MT862886	Dengue virus 1	V	2018-05-04
FIOCRUZ-0022	PB-711	MT862884	Dengue virus 1	V	2018-05-04
FIOCRUZ-0018	PB-705	MT862892	Dengue virus 1	V	2018-05-07
FIOCRUZ-0020	PB-707	MT862854	Dengue virus 1	V	2018-05-07
FIOCRUZ-0021	PB-709	MT862885	Dengue virus 1	V	2018-05-09
FIOCRUZ-0037	PB-731	MT862876	Dengue virus 1	V	2018-05-12
FIOCRUZ-0033	PB-736	MT862864	Dengue virus 1	V	2018-05-13

FIOCRUZ-0038	PB-735	MT862875	Dengue virus 1	V	2018-05-13
FIOCRUZ-0007	PB-782	MT862879	Dengue virus 1	V	2018-05-18
FIOCRUZ-0010	PB-634	MT862889	Dengue virus 1	V	2018-05-21
FIOCRUZ-0002	PB-761	MT862871	Dengue virus 1	V	2018-05-26
FIOCRUZ-0003	PB-762	MT862881	Dengue virus 1	V	2018-05-26
FIOCRUZ-0001	PB-760	MT862880	Dengue virus 1	V	2018-05-27
FIOCRUZ-0008	PB-832	MT862878	Dengue virus 1	V	2018-06-01
FIOCRUZ-0006	PB-894	MT862888	Dengue virus 1	V	2018-06-03
FIOCRUZ-0028	PB-742	MT862882	Dengue virus 1	V	2018-06-04
FIOCRUZ-0035	PB-810	MT862874	Dengue virus 1	V	2018-06-07
FIOCRUZ-0034	PB-786	MT862856	Dengue virus 1	V	2018-06-10
FIOCRUZ-0042	PB-854	MT862877	Dengue virus 1	V	2018-06-16
FIOCRUZ-0009	PB-891	MT862863	Dengue virus 1	V	2018-06-17
FIOCRUZ-0005	PB-840	MT862872	Dengue virus 1	V	2018-06-20
FIOCRUZ-0032	PB-954	MT862873	Dengue virus 1	V	2018-10-01
FIOCRUZ-0023	PB-1005	MT862887	Dengue virus 1	V	2018-11-28
FIOCRUZ-0041	PB-1170	MT862858	Dengue virus 1	V	2019-03-19
FIOCRUZ-0024	PB-1127	MT862855	Dengue virus 1	V	2019-03-25
FIOCRUZ-0004	PB-1258	MT862859	Dengue virus 1	V	2019-05-06
FIOCRUZ-0025	PB-1287	MT862870	Dengue virus 1	V	2019-05-06
FIOCRUZ-0029	PB-215	MT862861	Dengue virus 1	V	2019-05-13
FIOCRUZ-0040	PB-221	MT862857	Dengue virus 1	V	2019-05-13
FIOCRUZ-0031	PB-1428	MT862865	Dengue virus 1	V	2019-05-29
FIOCRUZ-0027	PB-1453	MT862868	Dengue virus 1	V	2019-06-14
FIOCRUZ-0026	PB-1383	MT862869	Dengue virus 1	V	2019-06-18
FIOCRUZ-0030	PB-1423	MT862866	Dengue virus 1	V	2019-06-18
FIOCRUZ-0036	PB-1387	MT862867	Dengue virus 1	V	2019-06-18
FIOCRUZ-0039	PB-222	MT862860	Dengue virus 1	V	2019-05-13

country: state, city	host	sequencing method
Brazil: Sao Paulo, Ribeirao Preto	Homo sapiens	metagenomics; NovaSeq; 2x100bp
Brazil: Sao Paulo, Ribeirao Preto	Homo sapiens	metagenomics; NovaSeq; 2x100bp
Brazil: Sao Paulo, Ribeirao Preto	Homo sapiens	metagenomics; NovaSeq; 2x100bp
Brazil: Sao Paulo, Ribeirao Preto	Homo sapiens	metagenomics; NovaSeq; 2x100bp
Brazil: Sao Paulo, Ribeirao Preto	Homo sapiens	metagenomics; NovaSeq; 2x100bp
Brazil: Sao Paulo, Ribeirao Preto	Homo sapiens	metagenomics; NovaSeq; 2x100bp
Brazil: Sao Paulo, Ribeirao Preto	Homo sapiens	metagenomics; NovaSeq; 2x100bp
Brazil: Sao Paulo, Ribeirao Preto	Homo sapiens	metagenomics; NovaSeq; 2x100bp
Brazil: Sao Paulo, Ribeirao Preto	Homo sapiens	metagenomics; NovaSeq; 2x100bp
Brazil: Sao Paulo, Ribeirao Preto	Homo sapiens	metagenomics; NovaSeq; 2x100bp
Brazil: Sao Paulo, Ribeirao Preto	Homo sapiens	metagenomics; NovaSeq; 2x100bp
Brazil: Sao Paulo, Ribeirao Preto	Homo sapiens	metagenomics; NovaSeq; 2x100bp
Brazil: Sao Paulo, Ribeirao Preto	Homo sapiens	metagenomics; NovaSeq; 2x100bp
Brazil: Sao Paulo, Ribeirao Preto	Homo sapiens	metagenomics; NovaSeq; 2x100bp
Brazil: Sao Paulo, Ribeirao Preto	Homo sapiens	metagenomics; NovaSeq; 2x100bp
Brazil: Sao Paulo, Ribeirao Preto	Homo sapiens	metagenomics; NovaSeq; 2x100bp
Brazil: Sao Paulo, Ribeirao Preto	Homo sapiens	metagenomics; NovaSeq; 2x100bp
Brazil: Sao Paulo, Ribeirao Preto	Homo sapiens	metagenomics; NovaSeq; 2x100bp
Brazil: Sao Paulo, Ribeirao Preto	Homo sapiens	metagenomics; NovaSeq; 2x100bp
Brazil: Sao Paulo, Ribeirao Preto	Homo sapiens	metagenomics; NovaSeq; 2x100bp
Brazil: Sao Paulo, Ribeirao Preto	Homo sapiens	metagenomics; NovaSeq; 2x100bp
Brazil: Sao Paulo, Ribeirao Preto	Homo sapiens	metagenomics; NovaSeq; 2x100bp
Brazil: Sao Paulo, Monte Aprazivel	Homo sapiens	metagenomics; NovaSeq; 2x100bp
Brazil: Sao Paulo, Ribeirao Preto	Homo sapiens	metagenomics; NovaSeq; 2x100bp
Brazil: Sao Paulo, Ribeirao Preto	Homo sapiens	metagenomics; NovaSeq; 2x100bp
Brazil: Sao Paulo, Ribeirao Preto	Homo sapiens	metagenomics; NovaSeq; 2x100bp
Brazil: Sao Paulo, Ribeirao Preto	Homo sapiens	metagenomics; NovaSeq; 2x100bp
Brazil: Paraiba, Coremas	Homo sapiens	PCR amplicon; MiSeq; 2x150bp
Brazil: Paraiba, Coremas	Homo sapiens	PCR amplicon; MiSeq; 2x150bp
Brazil: Paraiba, Coremas	Homo sapiens	PCR amplicon; MiSeq; 2x150bp
Brazil: Paraiba, Coremas	Homo sapiens	PCR amplicon; MiSeq; 2x150bp
Brazil: Paraiba, Coremas	Homo sapiens	PCR amplicon; MiSeq; 2x150bp
Brazil: Paraiba, Coremas	Homo sapiens	PCR amplicon; MiSeq; 2x150bp
Brazil: Paraiba, Coremas	Homo sapiens	PCR amplicon; MiSeq; 2x150bp
Brazil: Paraiba, Coremas	Homo sapiens	PCR amplicon; MiSeq; 2x150bp
Brazil: Paraiba, Coremas	Homo sapiens	PCR amplicon; MiSeq; 2x150bp
Brazil: Paraiba, Coremas	Homo sapiens	PCR amplicon; MiSeq; 2x150bp
Brazil: Paraiba, Coremas	Homo sapiens	PCR amplicon; MiSeq; 2x150bp
Brazil: Paraiba, Coremas	Homo sapiens	PCR amplicon; MiSeq; 2x150bp
Brazil: Paraiba, Sao Domingos	Homo sapiens	PCR amplicon; MiSeq; 2x150bp
Brazil: Paraiba, Pombal	Homo sapiens	PCR amplicon; MiSeq; 2x150bp

Brazil: Paraiba, Sao Domingos	Homo sapiens	PCR amplicon; MiSeq; 2x150bp
Brazil: Paraiba, Caturite	Homo sapiens	PCR amplicon; MiSeq; 2x150bp
Brazil: Paraiba, Coremas	Homo sapiens	PCR amplicon; MiSeq; 2x150bp
Brazil: Paraiba, Barauna	Homo sapiens	PCR amplicon; MiSeq; 2x150bp
Brazil: Paraiba, Barauna	Homo sapiens	PCR amplicon; MiSeq; 2x150bp
Brazil: Paraiba, Barauna	Homo sapiens	PCR amplicon; MiSeq; 2x150bp
Brazil: Paraiba, Caturite	Homo sapiens	PCR amplicon; MiSeq; 2x150bp
Brazil: Paraiba, Campina Grande	Homo sapiens	PCR amplicon; MiSeq; 2x150bp
Brazil: Paraiba, Joao Pessoa	Homo sapiens	PCR amplicon; MiSeq; 2x150bp
Brazil: Paraiba, Queimadas	Homo sapiens	PCR amplicon; MiSeq; 2x150bp
Brazil: Paraiba, Queimadas	Homo sapiens	PCR amplicon; MiSeq; 2x150bp
Brazil: Paraiba, Uirauna	Homo sapiens	PCR amplicon; MiSeq; 2x150bp
Brazil: Paraiba, Caturite	Homo sapiens	PCR amplicon; MiSeq; 2x150bp
Brazil: Paraiba, Campina Grande	Homo sapiens	PCR amplicon; MiSeq; 2x150bp
Brazil: Paraiba, Olivedos	Homo sapiens	PCR amplicon; MiSeq; 2x150bp
Brazil: Paraiba, Joao Pessoa	Homo sapiens	PCR amplicon; MiSeq; 2x150bp
Brazil: Paraiba, Sao Sebastiao Do Um	Homo sapiens	PCR amplicon; MiSeq; 2x150bp
Brazil: Paraiba, Joao Pessoa	Homo sapiens	PCR amplicon; MiSeq; 2x150bp
Brazil: Paraiba, Bayeux	Homo sapiens	PCR amplicon; MiSeq; 2x150bp
Brazil: Paraiba, Joao Pessoa	Homo sapiens	PCR amplicon; MiSeq; 2x150bp
Brazil: Alagoas, Maceio	Homo sapiens	PCR amplicon; MiSeq; 2x150bp
Brazil: Alagoas, Sao Miguel Dos Milag	Homo sapiens	PCR amplicon; MiSeq; 2x150bp
Brazil: Paraiba, Monteiro	Homo sapiens	PCR amplicon; MiSeq; 2x150bp
Brazil: Paraiba, Joao Pessoa	Homo sapiens	PCR amplicon; MiSeq; 2x150bp
Brazil: Paraiba, Joao Pessoa	Homo sapiens	PCR amplicon; MiSeq; 2x150bp
Brazil: Paraiba, Monteiro	Homo sapiens	PCR amplicon; MiSeq; 2x150bp
Brazil: Paraiba, Santa Rita	Homo sapiens	PCR amplicon; MiSeq; 2x150bp
Brazil: Alagoas, Sao Jose Da Laje	Homo sapiens	PCR amplicon; MiSeq; 2x150bp

number of sequencing reads	average depth of coverage	% genome covered	URL
456,512	5446.3	99.1	https://www.ncbi.nlm.nih.gov/nuccore/MW208056
19,466	213.8	77.8	https://www.ncbi.nlm.nih.gov/nuccore/MW208042
25,182	274	99.7	https://www.ncbi.nlm.nih.gov/nuccore/MW208043
19,862	213.3	97.4	https://www.ncbi.nlm.nih.gov/nuccore/MW208044
2,420	26	72	https://www.ncbi.nlm.nih.gov/nuccore/MW208053
5,088	56.1	79.8	https://www.ncbi.nlm.nih.gov/nuccore/MW208054
99,723	1104.9	98.9	https://www.ncbi.nlm.nih.gov/nuccore/MW208055
9,406	99.4	92.9	https://www.ncbi.nlm.nih.gov/nuccore/MW208057
35,581	386	98.3	https://www.ncbi.nlm.nih.gov/nuccore/MW208058
39,106	429	98.6	https://www.ncbi.nlm.nih.gov/nuccore/MW208059
16,069	140.4	96.4	https://www.ncbi.nlm.nih.gov/nuccore/MW208060
2,965	31.5	70.8	https://www.ncbi.nlm.nih.gov/nuccore/MW208041
7,659	87.6	83.5	https://www.ncbi.nlm.nih.gov/nuccore/MW208045
5,186	57.1	89.4	https://www.ncbi.nlm.nih.gov/nuccore/MW208046
4,951	53.3	81	https://www.ncbi.nlm.nih.gov/nuccore/MW208040
14,032	138,3	92.2	https://www.ncbi.nlm.nih.gov/nuccore/MW208047
6,092	64.3	85.9	https://www.ncbi.nlm.nih.gov/nuccore/MW208048
6,147	65.9	88.2	https://www.ncbi.nlm.nih.gov/nuccore/MW208049
31,202	355	98.6	https://www.ncbi.nlm.nih.gov/nuccore/MW208050
57,736	678.8	96.6	https://www.ncbi.nlm.nih.gov/nuccore/MW208051
4,548	51.5	78.3	https://www.ncbi.nlm.nih.gov/nuccore/MW208052
5,101	53.9	85.6	https://www.ncbi.nlm.nih.gov/nuccore/MW208062
40,586	439.6	98.2	https://www.ncbi.nlm.nih.gov/nuccore/MW208063
4,883	54.6	75.9	https://www.ncbi.nlm.nih.gov/nuccore/MW208061
4,703	51.6	86	https://www.ncbi.nlm.nih.gov/nuccore/MW208064
1,704	15	72.4	https://www.ncbi.nlm.nih.gov/nuccore/MW208065
3,372	20	92.7	https://www.ncbi.nlm.nih.gov/nuccore/MW208066
2035531	14182.62	99.86	https://www.ncbi.nlm.nih.gov/nuccore/MT862895
637672	4476.98	99.85	https://www.ncbi.nlm.nih.gov/nuccore/MT862893
990924	6988.85	99.97	https://www.ncbi.nlm.nih.gov/nuccore/MT862891
378233	2886.31	25.1	https://www.ncbi.nlm.nih.gov/nuccore/MT862862
1355348	9470.34	99.92	https://www.ncbi.nlm.nih.gov/nuccore/MT862890
946407	6663.52	99.85	https://www.ncbi.nlm.nih.gov/nuccore/MT862894
409018	2909.51	95.53	https://www.ncbi.nlm.nih.gov/nuccore/MT862883
1091826	7600.57	98.31	https://www.ncbi.nlm.nih.gov/nuccore/MT862886
1484703	10364.47	99.98	https://www.ncbi.nlm.nih.gov/nuccore/MT862884
1495585	10398.35	99.87	https://www.ncbi.nlm.nih.gov/nuccore/MT862892
1502978	10540.83	96.86	https://www.ncbi.nlm.nih.gov/nuccore/MT862854
1666274	11590.85	99.98	https://www.ncbi.nlm.nih.gov/nuccore/MT862885
2433007	16662.28	93.85	https://www.ncbi.nlm.nih.gov/nuccore/MT862876
2512589	17212.21	83.51	https://www.ncbi.nlm.nih.gov/nuccore/MT862864

1304395	9006.05	97.95	https://www.ncbi.nlm.nih.gov/nuccore/MT862875
879880	6163.87	99.88	https://www.ncbi.nlm.nih.gov/nuccore/MT862879
945645	6617.01	99.97	https://www.ncbi.nlm.nih.gov/nuccore/MT862889
2539383	17529.02	93.42	https://www.ncbi.nlm.nih.gov/nuccore/MT862871
2938246	20443.13	99.86	https://www.ncbi.nlm.nih.gov/nuccore/MT862881
1518806	10495.22	99.95	https://www.ncbi.nlm.nih.gov/nuccore/MT862880
1322953	9223.37	99.8	https://www.ncbi.nlm.nih.gov/nuccore/MT862878
5450932	38029.2	96.8	https://www.ncbi.nlm.nih.gov/nuccore/MT862888
1356750	9540.02	99.98	https://www.ncbi.nlm.nih.gov/nuccore/MT862882
1369827	9515.62	98.84	https://www.ncbi.nlm.nih.gov/nuccore/MT862874
2200445	15205.36	87.59	https://www.ncbi.nlm.nih.gov/nuccore/MT862856
2180170	14841.92	100	https://www.ncbi.nlm.nih.gov/nuccore/MT862877
3244005	22028.94	76.22	https://www.ncbi.nlm.nih.gov/nuccore/MT862863
2348727	16203.47	97.64	https://www.ncbi.nlm.nih.gov/nuccore/MT862872
1145093	7714.2	99.93	https://www.ncbi.nlm.nih.gov/nuccore/MT862873
800419	5478.18	99.97	https://www.ncbi.nlm.nih.gov/nuccore/MT862887
996730	6740.24	94.95	https://www.ncbi.nlm.nih.gov/nuccore/MT862858
860025	5832.29	93.52	https://www.ncbi.nlm.nih.gov/nuccore/MT862855
1091954	7507.61	95.09	https://www.ncbi.nlm.nih.gov/nuccore/MT862859
879085	5938.05	99.84	https://www.ncbi.nlm.nih.gov/nuccore/MT862870
962465	6771.71	92.35	https://www.ncbi.nlm.nih.gov/nuccore/MT862861
972032	6708.91	96.17	https://www.ncbi.nlm.nih.gov/nuccore/MT862857
1209833	8251.58	99.86	https://www.ncbi.nlm.nih.gov/nuccore/MT862865
2545868	17292.07	99.86	https://www.ncbi.nlm.nih.gov/nuccore/MT862868
1324626	9015.46	99.86	https://www.ncbi.nlm.nih.gov/nuccore/MT862869
1312119	8590.2	97.31	https://www.ncbi.nlm.nih.gov/nuccore/MT862866
1035378	7052.76	99.84	https://www.ncbi.nlm.nih.gov/nuccore/MT862867
1730526	11828.45	96.98	https://www.ncbi.nlm.nih.gov/nuccore/MT862860

Table S3. Metadata about age-stratified dengue virus seroprevalence surveys used to inform estimates of force of infection over time.

Study year	Authors	Age range	Region	Reference
2015	Chiaravalloti-Neto et al.	10-91	Southeast	1
1998	Teixeira et al.	All ages	Northeast	2
1998	Teixeira et al.	0-3	Northeast	3
1991	Vasconcelos et al.	All ages	North	4
1994	Vasconcelos et al.	All ages	Northeast	5
1994	Vasconcelos et al.	All ages	Northeast	6
1992	Moraes Figueiredo et al.	5-15	Southeast	7
1998	Lima et al.	All ages	Southeast	8
2001	Siqueira et al.	5-99	CentralWest	9
2002	Siqueira-Junior et al.	5-99	CentralWest	10
2007	Honório et al.	1-19	Southeast	11
2005	Braga et al.	5-64	Northeast	12
2006	Pessanha et al.	All ages	Southeast	13
2010	Martins et al.	0-12	North	14
1991	Cunha et al.	0-19	Southeast	15
1991	Cunha et al.	0-16	Southeast	16
2004	Muniz et al.	5-90	North	17

References

1 Chiaravalloti-Neto, F. et al. Seroprevalence for dengue virus in a hyperendemic area and associated socioeconomic and demographic factors using a cross-sectional design and a geostatistical approach, state of São Paulo, Brazil. BMC Infect. Dis. 19, 441 (2019).

2 Teixeira, M. da G. et al. Dynamics of dengue virus circulation: a silent epidemic in a complex urban area. Trop. Med. Int. Health 7, 757–762 (2002).

3 Teixeira, M. G. et al. Risk factors for the incidence of dengue virus infection in preschool children. Trop. Med. Int. Health 17, 1391–1395 (2012).

4 Vasconcelos, P. F. da C. et al. Epidemia de febre clássica de dengue causada pelo sorotipo 2 em Araguaiana, Tocantins, Brasil. Rev. Inst. Med. Trop. Sao Paulo 35, 141–148 (1993).

5 Vasconcelos, P. F. C. et al. Epidemia de dengue em Fortaleza, Ceará: inquérito soro-epidemiológico aleatório. Revista de Saúde Pública 32, 447–454 (1998).

6 Vasconcelos, P. F. et al. A seroepidemiological survey on the island of São Luis during a dengue epidemic in Maranhão. Rev. Soc. Bras. Med. Trop. 32, 171–179 (1999).

7 Moraes Figueiredo, L. T. et al. Encuesta serológica sobre el dengue en Ribeirao Preto, Sao Paulo, Brasil. Boletín de la Oficina Sanitaria Panamericana (OSP);118(6),jun. 1995 (1995).

8 Lima, V. L. C. de et al. Dengue: inquérito populacional para pesquisa de anticorpos e vigilância virológica no Município de Campinas, São Paulo, Brasil. Cadernos de Saúde Pública 23, 669–680 (2007).

9 Siqueira, J. B. et al. Household survey of dengue infection in central Brazil: spatial point pattern analysis and risk factors assessment. Am. J. Trop. Med. Hyg. 71, 646–651 (2004).

10 Siqueira-Junior, J. B. et al. Spatial point analysis based on dengue surveys at household level in central Brazil. BMC Public Health 8, 361 (2008).

11 Honório, N. A. et al. Spatial evaluation and modeling of Dengue seroprevalence and vector density in Rio de Janeiro, Brazil. PLoS Negl. Trop. Dis. 3, e545 (2009).

12 Braga, C. et al. Seroprevalence and risk factors for dengue infection in socio-economically distinct areas of Recife, Brazil. Acta Trop. 113, 234–240 (2010).

13 Pessanha, J. E. M., Caiaffa, W. T., Kroon, E. G. & Proietti, F. A. Dengue fever in three sanitary districts in the city of Belo Horizonte, Brazil: a population-based seroepidemiological survey, 2006 to 2007. Rev. Panam. Salud Publica 27, 252–258 (2010).

14 Martins, A. C. et al. Seroprevalence and seroconversion of dengue and implications for clinical diagnosis in amazonian children. Interdiscip. Perspect. Infect. Dis. 2014, 703875 (2014).

15 Cunha, R. V. da et al. Secondary dengue infection in schoolchildren in a dengue endemic area in the State of Rio de Janeiro, Brazil. Rev. Inst. Med. Trop. Sao Paulo 37, 517–521 (1995).

16 da Cunha, R. V. et al. Dengue infection in Paracambi, State of Rio de Janeiro, 1990-1995. Rev. Soc. Bras. Med. Trop. 30, 379–383 (1997).

17 da Silva-Nunes, M. et al. Risk factors for dengue virus infection in rural Amazonia: population-based cross-sectional surveys. Am. J. Trop. Med. Hyg. 79, 485–494 (2008).

Table S4. Priors for Index P Estimates

Parameter	Prior distribution	Sources
Human Incubation Period	Gaussian (mean = 5, SD = 1)	1–3
Human Infectious Period	Gaussian (mean = 5, SD = 1)	1–3
Human Life Expectancy	Gaussian (mean = 73, SD = 2)	4
Transmission probability (human to mosquito)	Gaussian (mean = 0.5, SD = 0.01)	5
Mosquito Biting Rate	Gaussian (mean = 0.25, SD = 0.05)	6,7
Extrinsic Incubation Period	Gaussian (mean = 7, SD = 2)	2,8,9
Mosquito Life Expectancy	Gaussian (mean = 14, SD = 3)	10,11

1 Lourenço J, de Lima MM, Faria NR, Walker A, Kraemer MUG, Villabona-Arenas CJ, et al. Epidemiological and ecological determinants of Zika virus transmission in an urban setting. eLife. 2017. doi:10.7554/elife.29820

2 Ferguson NM, Cucunubá ZM, Dorigatti I, Nedjati-Gilani GL, Donnelly CA, Basáñez M-G, et al. EPIDEMIOLOGY. Countering the Zika epidemic in Latin America. Science. 2016;353: 353–354.

3 Lessler J, Ott CT, Carcelen AC, Konikoff JM, Williamson J, Bi Q, et al. Times to key events in Zika virus infection and implications for blood donation: a systematic review. Bull World Health Organ. 2016;94: 841–849.

4 Life expectancy at birth, total (years) - Brazil | Data. [cited 13 Jul 2020]. Available: https://data.worldbank.org/indicator/SP.DYN.LE00.IN?locations=BR

5 Obolski U, Perez PN, Villabona-Arenas CJ, Thézé J, Faria NR, Lourenço J. MVSE : An R-package that estimates a climate-driven mosquito-borne viral suitability index. Poisot T, editor. Methods Ecol Evol. 2019;10: 1357–1370.

6 Yasuno M, Tonn RJ. A study of biting habits of Aedes aegypti in Bangkok, Thailand. Bull World Health Organ. 1970;43: 319–325.

7 Trpis M, Hausermann W. Dispersal and other population parameters of Aedes aegypti in an African village and their possible significance in epidemiology of vector-borne diseases. Am J Trop Med Hyg. 1986;35: 1263–1279.

8 Li MI, Wong PSJ, Ng LC, Tan CH. Oral susceptibility of Singapore Aedes (Stegomyia) aegypti (Linnaeus) to Zika virus. PLoS Negl Trop Dis. 2012;6: e1792.

9 Wong P-SJ, Li M-ZI, Chong C-S, Ng L-C, Tan C-H. Aedes (Stegomyia) albopictus (Skuse): a potential vector of Zika virus in Singapore. PLoS Negl Trop Dis. 2013;7: e2348.

10 Trpis M, Hausermann W, Craig GB. Estimates of Population Size, Dispersal, and Longevity of Domestic Aedes aegypti aegypti (Diptera: Culicidae) by Mark–Release–Recapture in the Village of Shauri Moyo in Eastern Kenya. Journal of Medical Entomology. 1995. pp. 27–33. doi:10.1093/jmedent/32.1.27

11 Hugo LE, Jeffery JAL, Trewin BJ, Wockner LF, Nguyen TY, Nguyen HL, et al. Adult survivorship of the dengue mosquito Aedes aegypti varies seasonally in central Vietnam. PLoS Negl Trop Dis. 2014;8: e2669.

Table S5. Tiled PCR primers used for DENV-1 amplicon-based sequencing.

Primer	Sequence	Primer Pool
DENV1SA_1_LEFT	TACGTGGACCGACAAGAACAGT	pool 1
DENV1SA_1_RIGHT	ACTATCATRTGTGGCTCTCCCC	pool 1
DENV1SA_2_LEFT	TGTTGAACATAATRAACAGGAGGAAAAGA	pool 2
DENV1SA_2_RIGHT	GAATCCTGGGTGTCKCAAAGCC	pool 2
DENV1SA_3_LEFT	CACACGTGGGACTTGGTCTAGA	pool 1
DENV1SA_3_RIGHT	ACACACAAAGTTCGCGTCTTGT	pool 1
DENV1SA_4_LEFT	ACTGTGCATTGAAGCTAAAATATCAAACA	pool 2
DENV1SA_4_RIGHT	ACCATTGTTTGTGGACAAGCCA	pool 2
DENV1SA_5_LEFT	CCTCACATTGGACTGCTCACCT	pool 1
DENV1SA_5_RIGHT	TGCACTARRACAGTTCCATGCT	pool 1
DENV1SA_6_LEFT	AAACTGACYTTARAGGGGATGTCAT	pool 2
DENV1SA_6_RIGHT	ATATGCRGTCCCAAAAACCTGG	pool 2
DENV1SA_7_LEFT	CGAGGAGCACGAAGGATGGC	pool 1
DENV1SA_7_RIGHT	ATGATGTTCTCAAGACGCGTGG	pool 1
DENV1SA_8_LEFT	AGGCTGACTCCCCAAAAAGACT	pool 2
DENV1SA_8_RIGHT	TTGATGGCAGCTGACATTAGCC	pool 2
DENV1SA_9_LEFT	TGGGAAGTTGAGGACTAYGGGT	pool 1
DENV1SA_9_RIGHT	TGTRGTTCTGAGRGATGGACCTC	pool 1
DENV1SA_10_LEFT	GCAGGGCCATGGCACCTAGG	pool 2
DENV1SA_10_RIGHT	TCCCCATCCTGTCTGAAGCATT	pool 2
DENV1SA_11_LEFT	GATGACTGGAACACTGGCTGTT	pool 1
DENV1SA_11_RIGHT	CACCGGAAGCCATGTTGTTTTT	pool 1
DENV1SA_12_LEFT	GGATTATGCATGGAARACAAYGGC	pool 2
DENV1SA_12_RIGHT	GTGAGTGTRTCATCCCTYTCTTCA	pool 2
DENV1SA_13_LEFT	AASAAGAAGCAGAACACTCCGG	pool 1
DENV1SA_13_RIGHT	ACTGGCCCAGCTTGGTTCCAG	pool 1
DENV1SA_14_LEFT	AGGTCCCAAGTAGGAGTGGGAGT	pool 2
DENV1SA_14_RIGHT	CACCTCRTCCTCAATCTCTGGT	pool 2
DENV1SA_15_LEFT	ATGGAGTGGTGACAACAAGTGG	pool 3
DENV1SA_15_RIGHT	GCTGGATCGGTAAARTGTGCTTC	pool 3
DENV1SA_16_LEFT	GGGAGATAGTTGACCTCATGTGCCA	pool 4
DENV1SA_16_RIGHT	CCTGTCGGCCCGGAAATTTGC	pool 4
DENV1SA_17_LEFT	ACGGGTRATYCAAYTGAGCAGRA	pool 3
DENV1SA_17_RIGHT	CCTCTTCTCATGAGCTCCACA	pool 3
DENV1SA_18_LEFT	CAGAAGGGATCATCCCAGCCCT	pool 4
DENV1SA_18_RIGHT	CCTCCTTGTTCGGAATTGTGCA	pool 4
DENV1SA_19_LEFT	AGTGTCTCAGGTGACCTAATATTGGA	pool 3
DENV1SA_19_RIGHT	RGCTGCCACTGTCAGTATCATG	pool 3
DENV1SA_20_LEFT	GCTGCTCATTCCAGARCCAGAC	pool 4
DENV1SA_20_RIGHT	ATGGGTTCACCTGGGAATAGCA	pool 4
DENV1SA_21_LEFT	YGCAAAYCAGGCWGCYATATTGAT	pool 3
DENV1SA_21_RIGHT	GATGTTTGCCATGGACACTGCT	pool 3
DENV1SA_22_LEFT	TCCATCACACTGGCTACTGGAC	pool 4

DENV1SA_22_RIGHT	CCCACAACCGAGGTCTATGACT	pool 4
DENV1SA_23_LEFT	ACAACCAAACATGCAGTGTCGA	pool 3
DENV1SA_23_RIGHT	TTTCGCACTAGCATCCCTCCAT	pool 3
DENV1SA_24_LEFT	GCTYAGAGGAAACCAATTCTGCA	pool 4
DENV1SA_24_RIGHT	TGATCCTGATGYTTGACCTCA	pool 4
DENV1SA_25_LEFT	ACCTAGATATYATTGGCCAGAGGA	pool 3
DENV1SA_25_RIGHT	ACCTTTCGTCTTCCACTGCTTC	pool 3
DENV1SA_26_LEFT	CTGCACAAGAGAGGAGTTCACA	pool 4
DENV1SA_26_RIGHT	TATTCTTGTGTCCCATCCGGCT	pool 4
DENV1SA_27_LEFT	TGGAAGGAGAAGGACTGCACAA	pool 3
DENV1SA_27_RIGHT	CACRCAATCATCTCCGCTRATT	pool 3
DENV1SA_28_LEFT	GAAACCCCCAAYCTAGCTRAGA	pool 4
DENV1SA_28_RIGHT	TAGCCGCTAGTCTCAGGTCTCT	pool 4
DENV1SA_29_LEFT	ATGGAGCCTGAGAGAAACTGCT	pool 3
DENV1SA_29_RIGHT	GCYCCTTCGGGATCACTCTCAT	pool 3
DENV1SA_30_LEFT	GGGCCACYAATATACAAGTAGCCA	pool 4
DENV1SA_30_RIGHT	CCCGCTGCTGCGTTATGTCT	pool 4
DENV1SA_31_RIGHT	CCTGTTGATTCAACAGCACCATTCCA	pool 4

Table S6. Log marginal likelihood estimates of the different clock models, as estimated using path-sampling stepping-stone sampling in BEAST v1.10.4. For each dataset we found that uncorrelated lognormal relaxed clock models fit better our data.

Alignment	Molecular clock model	PS	SS
DENV-1	Strict clock	-52476.12	-52483.72
DENV-2	Strict clock	-52752.40	-55768.42
DENV-1	Relaxed clock	-52438.27	-52452.42
DENV-2	Relaxed clock	-55551.05	-55563.21
