# Supplementary material for: Lying in wait: the resurgence of dengue virus after the Zika epidemic in Brazil
Source: Nat Commun. 2021 May 11;12:2619. doi: 10.1038/s41467-021-22921-7 (PMC8113494; doi:10.1038/s41467-021-22921-7)
Supplement: Supplementary file 3 — Reporting Summary [file 41467_2021_22921_MOESM3_ESM.pdf]

nature research

Corresponding author(s): Nathan Grubaugh

Last updated by author(s): Mar 29, 2021

Reporting Summary

Nature Research wishes to improve the reproducibility of the work that we publish. This form provides structure for consistency and transparency in reporting. For further information on Nature Research policies, see Authors & Referees and the Editorial Policy Checklist .

Statistics

For all statistical analyses, confirm that the following items are present in the figure legend, table legend, main text, or Methods section.

n/a	Confirmed	
☐	☒	The exact sample size (*n*) for each experimental group/condition, given as a discrete number and unit of measurement
☒	☐	A statement on whether measurements were taken from distinct samples or whether the same sample was measured repeatedly
☒	☐	The statistical test(s) used AND whether they are one- or two-sided<br>*Only common tests should be described solely by name; describe more complex techniques in the Methods section.*
☐	☒	A description of all covariates tested
☒	☐	A description of any assumptions or corrections, such as tests of normality and adjustment for multiple comparisons
☐	☒	A full description of the statistical parameters including central tendency (e.g. means) or other basic estimates (e.g. regression coefficient) AND variation (e.g. standard deviation) or associated estimates of uncertainty (e.g. confidence intervals)
☒	☐	For null hypothesis testing, the test statistic (e.g. *F*, *t*, *r*) with confidence intervals, effect sizes, degrees of freedom and *P* value noted<br>*Give P values as exact values whenever suitable.*
☐	☒	For Bayesian analysis, information on the choice of priors and Markov chain Monte Carlo settings
☒	☐	For hierarchical and complex designs, identification of the appropriate level for tests and full reporting of outcomes
☒	☐	Estimates of effect sizes (e.g. Cohen's *d*, Pearson's *r*), indicating how they were calculated

Our web collection on statistics for biologists contains articles on many of the points above.

Software and code

Policy information about availability of computer code

Data collection	No software was used.
Data analysis	Mosquito transmission potential was estimated using the R package MVSE (v.1); multiplex PCR primers covering the entire DENV-1 genomes were designed using PrimalScheme (v.1); aligned reads were filtered and output as a .bam file using SAMtools (version 1.9); Sorted .bam files containing only mapped, paired reads were visualized in Geneious Prime (version 2019.0.3) and consensus genomes were generated using the same software; genome sequences were aligned using MAFFT (v7.471); preliminary maximum-likelihood (ML) analyses were performed using IQTree (v.1.6.12); Genomes were inspected for the identification of major molecular clock outliers using TempEst (v.1.5.3); Bayesian phylogenetic inference was performed using BEAST (v.1.10.4), using the BEAGLE library (v3.1.0) to accelerate computation, and convergence of parameters was assessed using Tracer (v1.7); maximum clade credibility (MCC) trees were summarized using TreeAnnotator (v1.10.4), visualized using FigTree (v1.4.4), and plotted using baltic (v.0.1.0) and the R package Seraphim (no version available, retrieval date: 2019-11-18).

For manuscripts utilizing custom algorithms or software that are central to the research but not yet described in published literature, software must be made available to editors/reviewers. We strongly encourage code deposition in a community repository (e.g. GitHub). See the Nature Research guidelines for submitting code & software for further information.

Data

Policy information about availability of data

All manuscripts must include a data availability statement. This statement should provide the following information, where applicable:

- Accession codes, unique identifiers, or web links for publicly available datasets
- A list of figures that have associated raw data
- A description of any restrictions on data availability

The genomes generated in this study are available on NCBI (accession numbers: MT862854-MT862895; MW208040-MW208066), and listed in Table S2. Epidemiological data were downloaded from Brazil's Ministry of Health (http://www2.datasus.gov.br/DATASUS/index.php?area=0203&id=29878153), PAHO

website, and from the GitHub account of the Infectious Disease Dynamics Group at University of Florida (UF-IDD, https://github.com/UF-IDD/dengue-Zika-chik_Americas). All relevant data used in Figures 1, 2 and 4, 5 and 6 are available on the following GitHub repository: https://github.com/grubaughlab/DENV-genomics/tree/master/paper1.

Field-specific reporting

Please select the one below that is the best fit for your research. If you are not sure, read the appropriate sections before making your selection.

☐ Life sciences ☐ Behavioural & social sciences ☒ Ecological, evolutionary & environmental sciences

For a reference copy of the document with all sections, see nature.com/documents/nr-reporting-summary-flat.pdf

Ecological, evolutionary & environmental sciences study design

All studies must disclose on these points even when the disclosure is negative.

Study description	In this study we combined genomic, epidemiological, and ecological data to investigate the resurgence of dengue across Brazil and within two distinct geographic regions.
Research sample	Frozen serum from DENV-1 and DENV-2 positive patients were collected from two regions in Brazil: São Paulo state in the Southeast (DENV-1 and -2), and; Paraíba and Alagoas states in the Northeast (DENV-1). Age and gender of patients is not available, since the objective of this study was to analyze viral genomic data, and no human data was used.
Sampling strategy	No sample calculation was performed. All available samples from Paraiba and Alagoas states were obtained in distinct urban areas affected by DENV-1 in 2018-19. In Sao Paulo State samples were collected in two major urban areas in the central area, which were affected by DENV-2 in 2018-19. Some samples available from a 2010 DENV-2 outbreak in that state were also include to investigate if their origins are related to the latest outbreaks. For phylogeographic analyses, the larger and more geographically representative a dataset is, the better. Dengue samples are scarce, and we sampled as many samples as possible.
Data collection	None of the authors were present during sample collection. Samples were collected by healthcare workers in hospitals and sent to Public Health Labs. Concerning genomic data, they were collected manually from the online repositories ViPR and NCBI. The remaining genomes (n = 69) were generated by LCM, MJLS, JRF, RDOC, and CCK., using Nanopore and Illumina platforms.
Timing and spatial scale	Genomes of DENV-1 and DENV-2 sequences (full genomes and envelope sequences) were collected from all countries in the Americas were data were available, from 1977 to 2020. The newly sequenced full genomes were collected mostly in 2018-2019 from several urban areas, as described in 'Sampling strategy', due to their high dengue incidence, and according to their surveillance capacity.
Data exclusions	The exclusion criteria were pre-established. Genomes without collection dates and location information were excluded. Genomes from publicly available databases identified as outliers in root-to-tip analyses were removed from all phylogenetic analyses.
Reproducibility	The only experiment performed in this article was genome sequencing. We followed standard protocols (described in details in the Methodology), and each genome was sequenced only once, since the average depth of sequencing was large enough to provide good genome coverage (>70% coverage at 10X or higher).
Randomization	Randomization was not necessary. Phylogeographic analyses are more accurate as more data are included, and we included all available data, without performing any random selection beforehand.
Blinding	Blinding was not necessary in this study, since this is not a clinical research study.

Did the study involve field work? ☐ Yes ☒ No

Reporting for specific materials, systems and methods

We require information from authors about some types of materials, experimental systems and methods used in many studies. Here, indicate whether each material, system or method listed is relevant to your study. If you are not sure if a list item applies to your research, read the appropriate section before selecting a response.

Materials & experimental systems

n/a	Involved in the study
☒	☐ Antibodies
☒	☐ Eukaryotic cell lines
☒	☐ Palaeontology
☒	☐ Animals and other organisms
☒	☐ Human research participants
☒	☐ Clinical data

Methods

n/a	Involved in the study
☒	☐ ChIP-seq
☒	☐ Flow cytometry
☒	☐ MRI-based neuroimaging
